# Calpain proteolytic systems counteract endothelial cell adaptation to inflammatory environments

**DOI:** 10.1186/s41232-020-00114-x

**Published:** 2020-04-02

**Authors:** Takuro Miyazaki, Risako Akasu, Akira Miyazaki

**Affiliations:** Department of Biochemistry, Showa University School of Medicine, 1-5-8 Hatanodai, Shinagawa-ku, Tokyo, 142-8555 Japan

**Keywords:** Proteostasis, Adhesion molecules, Adherens junctions, Wound repair, Atherosclerosis, Tumor angiogenesis, Retinopathy, Vascular remodeling

## Abstract

Vascular endothelial cells (ECs) make up the innermost surface of arteries, veins, and capillaries, separating the remaining layers of the vessel wall from circulating blood. Under non-inflammatory conditions, ECs are quiescent and form a robust barrier structure; however, exposure to inflammatory stimuli induces changes in the expression of EC proteins that control transcellular permeability and facilitate angiogenic tube formation. Increasing evidence suggests that dysfunction in intracellular proteolytic systems disturbs EC adaptation to the inflammatory environment, leading to vascular disorders such as atherosclerosis and pathological angiogenesis. Recent work has highlighted the contribution of the calpain–calpastatin stress-responsive intracellular proteolytic system to adaptation failure in ECs. In this review, we summarize our current knowledge of calpain–calpastatin-mediated physiologic and pathogenic regulation in ECs and discuss the molecular basis by which disruption of this system perturbs EC adaptation to the inflammatory environment.

## Background

**Fig. 1:**
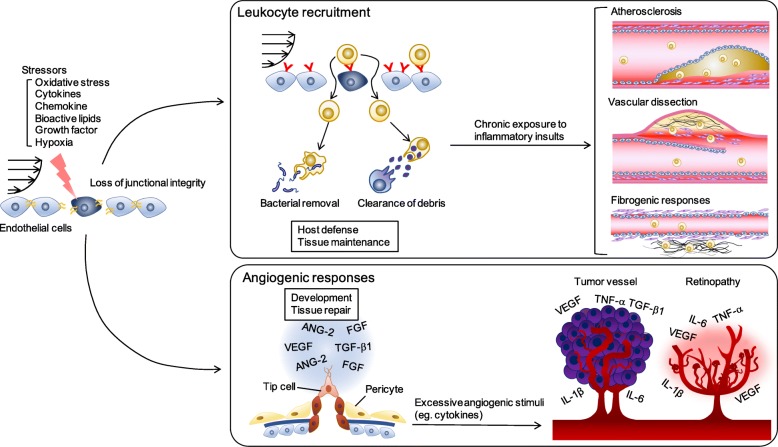
Endothelial adaptation to the inflammatory environment and related vascular disorders. Variety of stressors can elicit shedding of intercellular junctions in ECs (left). Loss of junctional integrity leads to recruitment of the leukocytes into the subendothelial spaces. While this adaptive event is required for the host defense and tissue maintenance, prolonged and excessive inflammatory responses cause failure of adaptation, thereby inducing fibrogenic responses in adjacent microenvironment as well as atherosclerosis and vascular dissection in large blood vessels (upper right). When ECs are subjected to the angiogenic stimuli, they migrate toward the interstitinal spaces to form new blood vessel network. Such angiogenic responses are indispensable for the tissue development and repair. However, excessive and redundant angiogenic stimuli, such as combination of growth factors and inflammatory cytokines, lead to aberrant angiogenic regulations, leading to the pathological angiogenesis, such as tumor angiogenesis and retinopathy (lower right). ANG-2: angiopoietin-2; EC: endothelial cell; FGF: fibroblast growth factor; IL-1β: interleukin-1β; IL-6: interleukin-6; TGF-β1: transforming growth factor-β1; TNF-α: tumor necrosis factor-α; VEGF: vascular endothelial growth factor

Vascular endothelial cells (ECs) form the luminal surface of blood vessels. Under non-inflammatory conditions, ECs are quiescent and form interendothelial junctions that prevent penetration of bioactive plasma components to the inner vascular wall [1]. Exposure to blood-borne stressors such as inflammatory cytokines, chemokines, growth factors, and bioactive lipids can activate ECs, triggering structural and biochemical responses that enable adaptation to the surrounding environment. For instance, many inflammatory cytokines increase the transcription of inflammatory adhesion molecules, such as selectins, intercellular adhesion molecule-1 (ICAM-1), and vascular cell adhesion molecule-1 (VCAM-1) [2, 3]. In turn, these changes promote adhesion of leukocytes to the luminal surface of ECs, and the subsequent recruitment of leukocytes into the subendothelial space. Concomitantly, interendothelial junctions are disrupted to release individual ECs, which further accelerates leukocyte recruitment [4], and fluid extravasation can lead to tissue edema [5]. Although leukocyte recruitment is required for host defense and tissue maintenance under normal conditions, excessive and prolonged leukocyte recruitment leads to vascular disorders, such as atherosclerosis, aneurysms, and fibrogenic responses. In addition to the junctional disorganization, activated ECs may exhibit outgrowth from the vessels and migrate toward a hypoxic environment, a process termed sprouting angiogenesis [6]. This event is mostly detected in the small vessels and is driven by several inflammatory cytokines and pro-angiogenic growth factors [6]. Postnatal angiogenesis is crucial for the normal maintenance and repair of tissues, but dysregulation of this process can contribute to pathologies such as tumor angiogenesis and diabetic retinopathy [6–8]. Thus, failure of ECs to correctly adapt to an inflammatory environment can cause a variety of vascular disorders (Fig. 1).

Recent studies from our group and others have shown that EC dysfunction can result from defective intracellular proteolytic systems. For instance, defects in autophagy in ECs can lead to aberrant regulation of cell survival, nitric oxide production, thrombogenicity, and angiogenesis [9]. Moreover, dysregulation of the ubiquitin–proteasome system can contribute to atherogenesis [10], defective angiogenesis [11], and insufficient production of vasomodulatory substances such as endothelin and nitric oxide [12]. In addition to autophagy and proteasomal function, calpain proteolytic systems have been shown to play a role in vascular regulation [13–15]. In this review, we summarize our current understanding of calpain-associated regulation of EC functions under inflammatory and non-inflammatory conditions, and we discuss the contribution of aberrant intracellular proteolysis to defective adaptation of ECs to the inflammatory environment.

## Calpain proteolytic systems in vascular endothelial cells

The calpain system consists of a family of calpain proteases and the endogenous calpain inhibitor calpastatin [16]. The mammalian calpain family contains 15 members classified as conventional and unconventional isozymes [17, 18]. The two conventional calpains, calpain-1 and calpain-2, require micromolar and millimolar Ca^2+^ concentrations, respectively, to achieve half-maximal activation [16–18]. Calpain-1 and calpain-2 are heterodimers consisting of a common regulatory subunit, *CAPNS1*, and a unique catalytic subunit, *CAPN1* and *CAPN2*, respectively [15]. These enzymes are ubiquitously expressed in ECs in the aorta [19], mesenteric microvasculature [20], pulmonary microvessels [21], and cardiac and brain capillaries [22, 23]. Early studies demonstrated that stressors and microRNAs can modulate calpain activity in ECs via changes in protein expression, or directly via changes in intracellular Ca^2+^ concentrations. For instance, hyperglycemia [24], thromboxane A2 [25], and lysophosphatidylcholine [21] have been reported to upregulate calpain expression in ECs, whereas the microRNAs miR-223 and miR-145 negatively regulate the expression levels of calpain through mRNA and non-mRNA-based mechanisms, respectively [26]. It was documented that treatment of spiral ganglion neurons with glutamic acid leads to elevation of mRNA levels of *Capn2* (calpain-2 gene) [27]; thus, the induction can be mediated through the transcriptional systems. While the overview of their transcriptional regulation is still sketchy, several researches identified a portion of the mechanisms. For instance, calpain induction by tumor necrosis factor-α can be reversed by p38-mitgen-activated protein kinase inhibitor SB203580 [28]; thus, the induction may be mediated through the p38 transcriptional systems. Other researchers documented that treatment of hepatocytes with CCl_4_ upregulates *Capn2* mRNA expression [29]. In this case, CCl_4_ stimulus concomitantly facilitated binding of AP-1 transcription factor complex to their binding motif, which is located upstream of *Capn2* gene. Hence, it is possible to consider that the expression levels of conventional calpains are modulated by a variety of inflammatory transcriptional systems. Conventional calpains are negatively regulated by calpastatin, which is facilitated by their subcellular colocalization in ECs, and not surprisingly, expression of calpastatin has been reported to be reduced in neovessels under pathological conditions [30]. Several growth factors, including epidermal growth factor, insulin-like growth factor, and vascular endothelial growth factor (VEGF), downregulate calpastatin expression in ECs [30]. These findings suggest that the calpain system is disinhibited in ECs during angiogenesis but is constitutively suppressed by calpastatin in quiescent ECs.

In contrast to the conventional calpains, the vasomodulatory roles of the unconventional calpains are unclear; nevertheless, these isozymes have been reported to have important functions under both physiological and pathological conditions. Mutation of calpain-3, which is exclusively expressed in skeletal muscle, contributes to limb-girdle muscular dystrophy type 2A [31]. G-calpain, which is a heterodimer of calpain-8 and calpain-9 expressed in the gastrointestinal tract, is associated with the prevention of gastric mucosal injury [32]. In addition, calpain-6, which lacks proteolytic activity due to a cysteine to lysine substitution in its active core, is upregulated in macrophages in atherosclerotic lesions, resulting in increasing cholesterol uptake in the cells and atherogenicity [33]. The reader is referred to several recent reviews for additional information on the unconventional calpains [16–18, 34, 35].

## Calpains play pivotal roles in the inflammatory properties of angiogenic endothelial cells

**Fig. 2:**
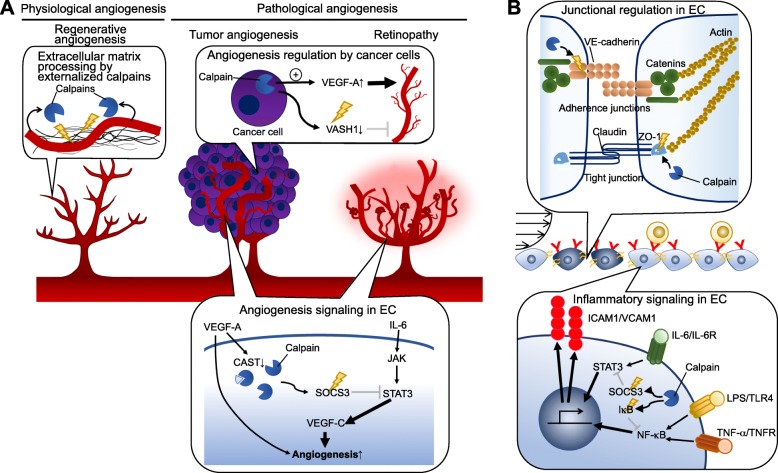
Calpain systems disturb EC adaptation to the inflammatory environment. **a** Conventional calpains can accelerate angiogenic responses in ECs. While conventional calpains externalized by ECs exert processing of extracellular matrix thereby accelerating regenerative angiogenesis, those in ECs can potentiate pathological angiogenesis, such as tumor angiogenesis and retinopathy. Mechanistically, calpain-induced proteolysis of SOCS3 accelerates cytokine-driven production of VEGF-C. VEGF-C can synergize with VEGF-A; thus, angiogenic responses in ECs can be excessive in the presence of redundant cytokine stimuli. Furthermore, conventional calpains in cancer cells degrade VASH1, an angiogenesis inhibitor, and potentiate the production of VEGF-A. Accordingly, calpains in cancer cells positively regulate tumor angiogenesis. **b** Conventional calpains can proteolyze the juxtamembrane domain of VE-cadherin, leading to the disorganization of adherence junctions in ECs. Furthermore, tight junction-associated protein ZO-1 can be degraded by calpain. As a result, overactivation of conventional calpains can disrupt junctional integrity in ECs. Concomitantly, calpain proteolyzes intracellular negative regulators of inflammatory signaling, such as IκB and SOCS3, in the cells. This amplifies cytokine responses in ECs, when the cells are subjected to the additional inflammatory stimuli. CAST: calpastatin; EC: endothelial cell; ICAM-1: intercellular adhesion molecule-1; IL-6: interleukin-6; IL-6R: interleukin-6 receptor; IκB: inhibitor κB; JAK: Janus kinase; LPS: lipopolysaccaride; NF-κB: nuclear factor-κB; STAT3: signal transducer and activator of transcription 3; SOCS3: suppressor of cytokine signaling 3; TLR4: toll-like receptor 4; TNF-α: tumor necrosis factor-α; TNFR: tumor necrosis factor receptor; VASH1: vasohibin-1; VCAM-1: vascular cell adhesion molecule-1; VE-cadherin: vascular endothelial-cadherin; VEGF: vascular endothelial growth factor; ZO-1; zonula occludens-1

While angiogenesis is a crucial process for normal physiology, it can also be pathological. Physiological angiogenesis occurs during tissue development and regeneration and is frequently driven through a pathway involving VEGF-A signaling and activation of the transcription factor hypoxia-inducible factor-1α (HIF-1α), which is primarily activated by hypoxia [6]. Conventional calpains have been associated with angiogenesis occurring during dermal wound repair [36], which occurs in a step-wise fashion involving sequential hemostasis, inflammation, proliferation, and remodeling processes [37]. Angiogenesis at the wound site appears to be initiated after the resolution of inflammation and occurs mainly during the proliferation and remodeling phases. Thus, dermal angiogenesis may not be influenced to any great extent by inflammatory stressors. Nassar et al. noted that transgenic mice overexpressing calpastatin displayed a delay in dermal wound repair accompanied by a reduced number of CD31^+^ blood vessels [36]. Interestingly, sections of wounded skin from wild-type mice grafted onto calpastatin transgenic mice also exhibited delayed wound repair and angiogenesis, indicating that calpain expressed in cells outside the wound site is necessary for both processes [36]. This is consistent with our previous finding that angiogenesis in wound sites is unaffected by EC-specific calpastatin overexpression, whereas wound repair is delayed [38]. Mechanistically, targeting EC calpain systems slows the differentiation and fibrogenic responses in adjacent myofibroblasts by impairment of platelet derived-growth factor-BB production [38]. Thus, the calpain system in ECs appears to influence the surrounding microenvironment rather than modifying the angiogenic properties of ECs themselves. Calpains also contribute to regenerative angiogenesis during the inflammatory resolution phase of glomerulonephritis [25]. Letavernier et al. noted that conventional calpains are externalized from ECs after exposure to VEGF-A or an adrenergic stimulus [39]. Externalized calpain appeared to mediate proteolysis of the extracellular matrix fibronectin to generate a 40-kDa breakdown product, leading to proliferation, migration, and angiogenic tube formation in ECs [39]. The fibronectin breakdown product was enriched in the kidney of mice with glomerulonephritis, but was present at lower levels in mice with transgenic overexpression of calpastatin [39]. It appears that calpains are released from cells through two distinct mechanisms: active secretion and leakage from deadly cells. Nishihara et al. previously documented that calpain-2, which is contained in the matrix vesicles, is released from unstimulated MC3T3-E1 cells [40]. Furthermore, calpain externalization from human umbilical ECs is reportedly potentiated by physiological stressors, such as VEGF and norepinephrine [39]. More recent, Hanouna et al. noted that calpain externalization is prevented by siRNA-based silencing of ABCA1; thus, those processes are probably driven through the molecular machinery [41]. Another possible mechanism underlying calpain externalization is a leakage from deadly cells. While conventional calpain, an 80-kDa protein, is unable to diffuse across the intact plasma membrane, injury of plasma membrane may increase its permeability. Indeed, Limaye et al. reported that externalized calpains, which are derived from deadly hepatocytes, exacerbates murine acute liver injury [42]. It is thought that the calpain leakage may have a substantial impact on the organ functions when the deadly calpain-expressing cells are highly accumulated in the surrounding microenvironment. Collectively, these observations indicated that extracellular calpains, or perhaps calpains expressed by non-EC cell types, modulate regenerative angiogenesis in ECs, while the direct contribution of the EC-endogenous calpain system is likely negligible (Fig. 2a).

In contrast to the physiological process, pathological angiogenesis is frequently detected in inflamed conditions, such as diabetic retinopathy and tumor growth, and reportedly exacerbates disease progression [6–8]. Inflammatory cytokines can elicit angiogenesis in ECs concomitantly with VEGF-A, and in some cases, they can disturb the angiogenic process. Notably, calpain systems have been associated with pathological angiogenesis. We previously showed that EC-specific overexpression of calpastatin did not affect vasculogenesis or vascular integrity in adult mice; however, it abrogated angiogenesis under pathological conditions such as tumor growth and oxygen-induced retinopathy [30]. Mechanistically, VEGF-A was shown to downregulate calpastatin in ECs, which facilitated degradation of suppressor of cytokine signaling 3 (SOCS3), an endogenous inhibitor of the Janus kinase–signal transducer and activator of transcription (JAK–STAT) signaling cascade [30]. Accordingly, loss of calpastatin sensitized the ECs to inflammatory cytokines, such as interleukin (IL)-6, leading to transcription of target genes, including VEGF-C. This growth factor is a well-known lymphangiogenic driver, and it appears to be able to elicit angiogenesis even in the presence of VEGF-A through VEGF receptor-2 and VEGF receptor-3 [43]. Indeed, VEGF-C is enriched in the cancer microenvironment and in the retinal lesions seen in retinopathy. Administration of a neutralizing anti-VEGF-C antibody has been shown to ameliorate oxygen-induced retinopathy [30]. Thus, conventional calpains appear to sensitize the JAK–STAT–VEGF-C axis in ECs to potentiate pathological angiogenesis (Fig. 2a). The contribution of conventional calpains to pathological angiogenesis has been confirmed in studies with pharmacological calpain inhibitors [44, 45], suggesting that conventional calpains may be promising therapeutic targets for the treatment of disorders associated with pathological angiogenesis.

In addition to their roles in ECs, conventional calpains expressed in other cell types can facilitate pathological angiogenesis in ECs through indirect mechanisms. Zheng et al. reported that calpain expressed in cancer cells proteolyzes filamin A, a large cytoskeletal actin-binding protein, to C-terminal fragments [46]. The fragments interact with HIF-1α during hypoxia, thereby promoting its nuclear translocation and subsequent transcription of target genes such as VEGF-A [46]. Thus, hypoxia-induced calpain activation in cancer cells enhances angiogenesis in the tumor microenvironment. Saito et al. noted that proteolytic degradation of vasohibin-1, a robust angiogenesis inhibitor, was enhanced in cancer cells [47], whereas calpains appeared to be stably activated, suggesting a mechanism for the reduction in vasohibin-1. Accordingly, pharmacological inhibition of calpains was shown to increase vasohibin-1 levels in the cancer cells, which led to a reduction in migration of adjacent ECs [47]. Collectively, these observations indicate that the calpain systems in ECs and other cells support the induction of angiogenesis in response to inflammatory stimuli (Fig. 2a).

Although the majority of studies support a pro-angiogenic role for calpains, a recent study has challenged that notion. Teng et al. noted that EC-specific downregulation of conventional calpains in mice exhibited accelerated myocardial angiogenesis in diabetic cardiomyopathy [22]. Angiogenic tube formation and migration of cardiac microvascular ECs in culture were interrupted by addition of palmitic acid, and this was rescued by overexpression of calpastatin [22]. The authors also demonstrated that calpain-2 proteolyzes β-catenin in these cells [22]. There are several potential explanations for the discrepancy in data from this study and others, including differences in cell type- and/or stressor-specific roles of the calpain system. Further investigations will be necessary to dissect the mechanisms underlying these results.

## Conventional calpains modulate the endothelial barrier

The EC monolayer forms interendothelial connections through adherens junctions, tight junctions, and gap junctions [48]. Among these, adherens junctions, which are ubiquitous from capillaries to the large vessels, are the most relevant to leukocyte recruitment into the subendothelial microenvironment [49–51]. Adherens junctions are formed by Ca^2+^-sensitive homophilic association between vascular endothelial–cadherins (VE-cadherins) and have been reported to be disorganized during atherogenesis [52, 53], predominantly through the action of calpain-2 [21]. Our previous study identified that exposure of ECs to lysophosphatidylcholine during the initial phase of atherosclerosis increases the expression of calpain-2, which proteolyzes the juxtamembrane domain of VE-cadherin, thereby destabilizing the adherens junction [21]. Such disorganization enhances the recruitment of circulating monocytes/macrophages into the subendothelial space. Accordingly, calpain overactivation in ECs exacerbates atherosclerosis. Su et al. have found that calpain-1 also mediates VE-cadherin proteolysis, thereby contributing to the intracellular trafficking of VE-cadherin to maintain adherens junctions [54]. VE-cadherin disorganization is also found in the pulmonary microvascular ECs in response to pressure overload [55]. In this case, the mechanosensitive ion channel Piezo1 activates the conventional calpains, which facilitates adherens junction disorganization and promotes pulmonary edema [55]. Scalia et al. found that albumin permeability is higher in the mesenteric postcapillary venules of Zucker diabetic rats compared with lean Zucker rats [20]. This leakage was reduced by administration of antisense oligonucleotides against calpain-1 or the pharmacological calpain inhibitor ZLLal. Taken together, these data indicate that conventional calpains proteolytically modulate the barrier functions of ECs in microvessels and large arteries via degradation of VE-cadherins and disruption of adherens junctions (Fig. 2b).

The blood–brain barrier is formed by pericytes, astrocytes, and ECs lining intracerebral capillaries [56]. Several lines of evidence suggest that conventional calpains contribute to pathological defects in the blood–brain barrier. Ischemia/reperfusion injury in rats induces calpain expression in peri-infarct penumbra cortex, resulting in hyperpermeability of cerebral microvessels and subsequent brain edema [23]. Tight junctions composed of occludin, claudin, junctional adhesion molecule-1, and zonula occludens-1 (ZO-1) play important roles in the barrier functions of cerebral microvascular ECs [57]. Alluri et al. noted that hyperpermeability of the blood–brain barrier induced by traumatic brain injury can be reversed by administration of calpain inhibitor III [58]. Based on immunocytochemical analyses, the authors suggested that treatment of rat brain microvascular ECs with IL-1β caused disorganization of ZO-1-associated tight junctions, which was inhibited by calpain inhibitor III treatment [58]. However, proteolysis of ZO-1 was undetectable in IL-1β-treated cells, suggesting that conventional calpains probably modulate tight junctions via proteolysis of other molecules within or outside the tight junction. Wang et al. showed that exposure of pulmonary ECs to particulate matter in polluted air caused hyperpermeability and increased the intracellular Ca^2+^ concentration via effects on the ion channel TRPM2 (transient receptor potential ion channels, subfamily M, member 2), leading to calpain activation and degradation of ZO-1 [59]. Although the mechanisms underlying the calpain-mediated barrier regulations in cerebral and pulmonary microvascular ECs are currently obscure, it is clear that calpain systems in ECs play crucial roles in the barrier regulations upon exposure to inflammatory and traumatic stressors (Fig. 2b).

## How does calpain interrupt EC adaptation to the inflammatory environment?

As noted above, conventional calpains influence EC functions during physiological angiogenesis driven by growth factors through mechanisms involving calpains externalized by ECs or expressed in other cell types, rather than through the EC-endogenous calpain system (Fig. 2a). Moreover, deficiency of calpain-1 and calpain-2 does not influence vessel formation during embryogenesis [60, 61], indicating that the angiogenic functions of calpains are not essential, at least during development. In contrast to growth factors, several bioactive lipids and inflammatory cytokines can activate endogenous conventional calpains in ECs, leading to junctional disorganization and the promotion of angiogenesis. Intriguingly, formation of adherens junction between ECs appears to limit their angiogenic ability [62]. Consistent with this, loss of VE-cadherin leads to activation of the phosphoinositide 3-kinase–AKT/Yes-associated protein–angiopoietin-2 signaling axis, which confers angiogenic properties on the cells [62]. Thus, it is possible that VE-cadherin disorganization induced by conventional calpains facilitates angiogenic responses in ECs and subsequent EC adaptation to the extracellular environment. Some intracellular negative regulators of inflammatory signaling, such as inhibitor-κB and SOCS3, are degraded by calpains in parallel with junctional disorganization (Fig. 2b), although the reasons for this are unclear. Thus, calpain activation sensitizes cytokine responses in ECs, when the cells are subjected to redundant inflammatory stimuli. The EC calpain system may thus be most important to the adaptation failure in the cells under severe inflammatory conditions, which is accomplished by sensitization of ECs to the environmental stressors, rather than by disturbing EC functions directly.

## Clinical perspective

Since the first-generation calpain inhibitors, such as leupeptin and E-64, were developed in the late 1960s and 1970s, many calpain inhibitors have been synthesized and tested in the biochemical and biomedical fields. Some of those agents are likely to be highly effective in treating pathophysiologic insults in animal models, including cardiovascular disease, neurodegenerative disease, ocular disease, cancer, and muscular dystrophy models [18]. Several pharmacological calpain inhibitors have been in preclinical and clinical testing for non-cardiovascular diseases, such as neurodegenerative diseases, spinal muscular atrophy, and multiple sclerosis [18]. For instance, ABT-957, a derivative of A-705239, was developed by AbbVie and had been subjected to phase I clinical trial for Alzheimer’s disease. On the other hand, it was reported that olesoxime, a mitochondrial-targeted neuroprotective compound, can suppress calpain activity in rat Huntington disease models [63, 64]. Olesoxime had been in clinical trials as a therapeutic agent for motor neuron diseases, such as relapsing–remitting multiple sclerosis, spinal muscular atrophy, and amyotrophic lateral sclerosis [18]. In contrast, calpain inhibitors were not served as a therapeutic agent for aforementioned vascular disorders, such as atherosclerosis, cancer (tumor neovessels), and retinopathy in clinical trials. Considering the animal data indicating that those vascular disorders can be suppressed by the calpain inhibition, calpain inhibitors are highly attractive for controlling those diseases. Although many compounds have been developed as a calpain inhibitor, target specificity of those agents among other proteases as well as calpain isozymes appears to be insufficient. Further studies will be necessary for improving the specificity of the inhibitors.

## Conclusions

Conventional calpains play a deleterious role during EC adaptation to the surrounding environment under severe inflammatory conditions, but they do not affect EC function under physiological conditions. This is an ideal scenario for a therapeutic target, making inhibition of the calpain system in ECs an attractive strategy. Several pharmacological calpain inhibitors have been subjected to the preclinical and clinical trials for non-cardiovascular diseases [18]. It will be of great interest to determine whether those agents can be repositioned for the treatment of cardiovascular disorders.

## Data Availability

None.

## Abbreviations

EC: Endothelial cells
HIF-1α: Hypoxia-inducible factor-1α
ICAM-1: Intercellular adhesion molecule-1
IL: Interleukin
JAK–STAT: Janus kinase–signal transducer and activator of transcription
SOCS3: Suppressor of cytokine signaling 3
VCAM-1: Vascular cell adhesion molecule-1
VE-cadherin: Vascular endothelial–cadherin
VEGF: Vascular endothelial growth factor
ZO-1: Zonula occludens-1
